# Qualitative Analysis of Maternal Barriers and Perceptions **t**o Participation in a Federal Supplemental Nutrition Program in Rural Appalachian North Carolina

**DOI:** 10.13023/jah.0204.06

**Published:** 2020-09-01

**Authors:** Sydeena E. Isaacs, Lenka Shriver, Lauren Haldeman

**Affiliations:** University of North Carolina at Greensboro, Department of Nutrition

**Keywords:** Appalachia, federal nutrition program, maternal barriers and perceptions, WIC experience, rural

## Abstract

**Background:**

Little is known about barriers to and perceptions of participation in the in Women, Infants, and Children (WIC) program in rural Appalachia.

**Purpose:**

To gain a deeper understanding of maternal barriers and perceptions related to WIC participation in rural Appalachia

**Methods:**

Pregnant women and mothers were recruited in-person and via flyers from WIC offices in three counties in Appalachian North Carolina. Four semi-structured focus groups were conducted between May to July 2018. Each focus group was approximately 60 minutes long and included open-ended questions about the overall WIC experience in rural Appalachia. Focus groups were audio-recorded, transcribed verbatim, and content analysis of transcripts was performed by two trained researchers. Identified themes were discussed and consensus was reached by the researchers to generate final themes for four areas of interest: (1) most valued aspects of WIC program, (2) barriers to program participation and benefit redemption, (3) experiences during appointments, and (4) suggestions for improving experiences in program.

**Results:**

The most valued aspects of participation were financial benefits and support/resources provided by WIC staff. In contrast, lack of variety of WIC-approved foods and social stigma were perceived as major barriers to participation and redeeming benefits.

**Implications:**

This study contributes to a better understanding of the barriers and perceptions related to WIC participation in this geographically and culturally unique area of rural Appalachia. Findings are valuable for informing WIC state-agencies and policymakers whose efforts focus on the identification and development of effective recruitment and retention strategies for WIC-eligible families in rural Appalachia.

## INTRODUCTION

Rural populations are disproportionately affected by many health inequalities compared to the rest of the nation, including a higher incidence of chronic diseases, higher mortality rates, and lower life expectancies.^1^ The Appalachian Region, also known as Appalachia, is one of the rural regions that experiences both significant socioeconomic and health inequalities compared to the rest of the nation.^2^ Appalachia encompasses 13 states from northern Mississippi to New York’s southern tier and is home to more than 25 million people.^3^ Notably, 42% of the region is rural. Currently, the region has higher mortality rates for seven of the leading causes of death nationwide (e.g., heart disease, stroke, diabetes), with all of them being influenced by lifestyle habits.^2^

Women, infants, and children living in rural communities are particularly vulnerable to these disparities.^4^ For example, women living in rural communities have poorer access to pre- and peri-natal care^5^ compared to their urban counterparts, and rural children are less likely to have preventive health care than children living in urban areas.^6^ Socioeconomic inequalities are also evident between rural and urban areas.^7^,^8^ Nearly one in four (24%) rural children live in poverty compared to one in five among urban children in the U.S. Lower socioeconomic status and rural residence are also associated with higher rates of food insecurity in both adults and children.^9^ Further, food insecurity in households with young children is inversely associated with diet quality.^10^,^11^ Thus, low-income families with young children living in rural communities represent a segment of the population most vulnerable to the negative consequences of health inequalities. Since growing evidence suggests that health disparities in Appalachia are widening, there is an urgent need to develop effective interventions to reduce health disparities and improve the overall well-being of rural families, especially among pregnant women, infants, and young children, as they are being negatively affected to the greatest degree.^12^

The Supplemental Nutrition Program for Women, Infants, and Children (WIC) is a federal nutrition program that serves more than seven million low-income pregnant and postpartum women, infants, and children nationwide.^13^ It is one of the nation’s most successful and cost-effective nutrition intervention programs, with 75% of those served by the program living in households below the federal poverty line.^14^ WIC provides a variety of benefits, ranging from nutrient-dense foods, nutrition education, breastfeeding support, and referrals to other healthcare providers.^13^ WIC participants tend to have greater access to health care and nutritious foods^13^ as well as improved pre- and post-natal maternal and infant health outcomes.^15^

Despite the WIC program’s benefits, the program has historically been underutilized.^16^ Participation rates have, in fact, declined nationwide from 63% of eligible families in 2011 to only 51% in 2017. Further, research suggests that some benefits are underutilized by geographic location, specifically in rural areas.^17^,^18^ WIC-eligible families living in rural communities represent a population in great need of federal nutrition assistance but face unique barriers and circumstances that inhibit participation in such programs. To date, research has identified a number of barriers to WIC participation across the nation, including difficulty scheduling an appointment or long wait times,^19^,^20^ lack of transportation,^20^–^22^ and confusion about program eligibility criteria.^19^,^20^,^23^ However, it is apparent that the perceptions and influences of these barriers on participation and retention vary by culture, ethnicity, marital status, family size, and/or geographic location.^16^,^22^

To date, participants’ attitudes and perceptions related to WIC program participation have not been examined in rural Appalachia. The primary aim of this study was to explore maternal attitudes, barriers, and perceptions of WIC participation in the Appalachian Region of North Carolina.

## METHODS

### Research Design and Participants

This study was part of larger formative research to identify potential intervention foci and develop initiatives to reduce WIC participation barriers and enhance program retention among low-income families in a three-county WIC agency in Appalachian North Carolina. The counties included in this study have stratified degrees of rurality (i.e., rural-urban continuum codes 5, 7, and 9) and population densities (56,000, 28,000, and 11,000, respectively).^24^–^26^ Median household incomes across the counties are 28%, 30%, and 32%, respectively, below the national average ($53,172). Likewise, rates of poverty are 73%, 35%, and 77%, respectively, higher than the national average (11.8%). The majority of residents in the three counties identify as non-Hispanic white, with 3.4%, 5.1%, and 10%, respectively, identifying as Hispanic/Latino. Two percent or less of residents in each county identify as black.

Pregnant women and mothers participating in the WIC program in these three counties were recruited in-person and via flyers. Participants were eligible for focus groups if they met the following inclusion criteria: (1) 18 years or older, (2) enrolled and/or has a child or children currently enrolled in the WIC program, (3) the primary person who redeems WIC food benefits/attends clinic visits, and (4) speaks English. Non-English speakers were excluded due to lack of a trained translator to conduct focus groups in another language. The study protocol and procedures were approved by the University of North Carolina’s Institutional Review Board.

### Procedures: Focus Group Recruitment

In each county, a researcher was on-site in the WIC clinics on two separate days for in-person recruitment. Since families receive WIC benefits on site only every 3 months, different days and times of the week were targeted to increase diversity of potential participants. If a participant met all eligibility criteria, the researcher obtained the participant’s contact information, and a focus group was scheduled via phone. When the researcher was not available for recruitment on-site, WIC clerks handed out recruitment flyers and interested participants were encouraged to contact the researcher directly via phone or email. Recruitment flyers were also posted in common areas of each health center (i.e., the lobby entrance). To further increase the diversity of participants recruited, WIC staff mailed recruitment flyers and letters along with other regular WIC mail to an additional 55 participants. A text message reminder was sent to all participants the day before the scheduled focus group to confirm their attendance.

### Data Collection

A focus group guide was developed specifically for the current study to explore participants’ perceptions and experiences related to their participation in the WIC program. The guide followed a semi-structured questionnaire format^27^ and was developed based on an extensive review of the literature^19^,^20^ and the needs of the WIC program, determined by the WIC director, at the time of the study. The question route was developed around four main areas of interest: (1) most valued aspects of the WIC program, (2) barriers related to participating in the program and redeeming WIC benefits, (3) the quality and nature of experiences during WIC appointments and (4) suggestions for improving experiences in the WIC program. Sample questions included “*Tell me about what motivates you to participate in the WIC program.”* and “*If you could change one thing about the WIC shopping experience, what would you change?”*

The guide was reviewed by three nutrition researchers with expertise in nutrition behavior in low-income families with children, one psychology researcher specialized in low-income families with children, and six WIC staff members who have daily experience with the program’s participants. The original guide was revised using the input and feedback from the reviewers.

Focus groups were conducted by a trained researcher at local public libraries or a hotel conference room. Each focus group lasted approximately 60 minutes and was audio-recorded. Participants reviewed an IRB-approved informed consent form and provided verbal consent upon their arrival to the focus group. Participants used only their first names throughout the focus group and filled out a brief questionnaire with sociodemographic information after the focus group. Participant incentives included a $20 gift card and light refreshments during the focus group.

Field notes were completed immediately following each focus group to document overall impressions, main themes discussed, and any other information that could be relevant for data analysis. Audio recordings were transcribed verbatim utilizing 2020 Temi, speech recognition software (https://www.temi.com/), and reviewed by the primary researcher to ensure accuracy and detail of the data.

### Data Analysis

Focus groups were conducted until themes reached congruence, the point at which no new themes emerged.^28^,^29^ Data were then analyzed using qualitative content analysis. Following a continuous, 3-step process adapted from previous research,^30^,^31^ two trained researchers used Atlas.ti, a qualitative data analysis software, to independently analyze and code focus group transcripts and identify common emerging themes. Initial “bracketing” and de-contextualization of the data was completed first.^32^ “Bracketing” is a widely utilized qualitative analysis approach that refers to the identification and suspension of any conjectures or ideas that may influence interpretation of the data, and it occurs continuously throughout the research process. During this step, researchers used the *highlight* function of Atlas.ti to identify keywords, phrases, and topics relevant to the research questions. These highlighted sections became *quotations* that were used for coding in Step 2. In Step 2, researchers independently coded the transcripts using a constant comparative, emergent coding design.^33^ Thematic categories from each construct of interest were identified and a preliminary code list was constructed. In the third step of content analysis, related codes and themes that emerged for each question were identified.^34^ Themes were summarized independently by the two researchers, then discussed until consensus was reached. Conceptual diagrams were generated during data analysis to provide context for each thematic category and to complete the exhaustive content analysis. Decisions were made about the final themes and a final code list was generated.^33^

## RESULTS

Four focus groups (n= 4 County 1; n= 2 and n= 3 County 2; n=6 County 3) were conducted with a total of 15 mothers and pregnant women. Demographic characteristics of the participants are presented in Table 1. The mean age of participants was 28.7 years. The majority of participants were non-Hispanic white (93%), lived in a household with at least two adults including themselves (87%), and had one or more children currently enrolled in the WIC program (87%). Nearly half of participants had a 4-year college degree or higher (Table 1). Years of experience participating in the WIC program ranged from 4 months to 9 years (data not shown), with the average length of experience 3.3 years across the sample. All nonpregnant participants in the sample fell into an overweight or obese category based on their body mass index (BMI) (9 of 12 were in the obese weight status category).

The content analysis revealed several themes that emerged during the focus groups around the four areas of interest. The main themes are described below and selected quotes for each theme are presented in Table 2 (see Additional Files).

### 1. Most Valued Aspects of WIC Participation

*Financial benefits* were the most valued aspects of WIC participation (Theme 1A). Participants noted that WIC helped offset the costs of buying groceries for their families and thus allows for the allocation of money to other household expenses. Provision of WIC-approved foods and/or formula and/or breastfeeding supplies (e.g., a breast pump) were also highly valued. All participants with breastfeeding experience reported they would not have been able to afford to purchase a breast pump without participating in WIC. Participants felt that WIC provided much more than “just food.” Referrals to other healthcare providers and other sources of food assistance were additional valuable *support/resource benefits* reported (Theme 1B). Prenatal and breastfeeding support and education were other frequently reported and highly valued nonfinancial benefits.

### 2. Experiences During WIC Appointments

The positive experiences were consistent across the district. Participants highly regarded the *efficiency of clinic visits*, noting the convenience and flexibility of scheduling a WIC appointment and short wait time to be seen (Theme 2A). A *caring and nurturing approach* by staff was another positive aspect of the WIC office experience (Theme 2B). Participants reported staff consistently answered their questions, addressed their concerns, and made them feel valued and heard. Several negative aspects of office visits also emerged. *Discrepancies in nutrition recommendations* between the WIC nutritionist and pediatricians were noted, although this theme was not consistent across all focus groups (Theme 2C). Coupling this discrepancy was the feeling that staff sometimes employed a *high-pressure approach* when providing nutrition education, which further contributed to a negative office experience (Theme 2D). Notably, participants reported feeling pressured by WIC staff to prove their ability to breastfeed and felt this practice could discourage participation in the program.

### 3. Barriers Related to Redeeming Food Benefits

Participants reported several barriers related to redeeming their food benefits. *Poor labeling* and *inconsistency/variability of WIC-approved items across grocery stores* were cited as major barriers (Theme 3A). Poor labeling included both a general lack of labeling in some stores and labeling with very small font in other stores, which made it difficult to identify WIC-approved items. Participants reported *problems redeeming some WIC-approved items* (i.e. peanut butter, bread) with issues varying by the grocery store (Theme 3B). General *lack of variety of WIC-approved foods locally available* to purchase (i.e. fruits and vegetables (FV) and whole grains) was another significant barrier (Theme 3C). Participants in the smaller and more rural counties reported the most limited variety and availability in their area. A *limited number of grocery stores* was an additional barrier discussed during the focus groups (Theme 3D). *Delays at checkout* due to having to separate WIC foods from non-WIC foods emerged as one of the biggest barriers to benefit redemption (Theme 3E). Participants noted the checkout experience frequently evoked feelings of anxiety and embarrassment, a direct result of *social stigma*, which emerged as the most frequently reported perceived barrier to WIC benefit redemption (Theme 3F). Participants felt that WIC and other federal and state assistance programs for those in need were generally perceived negatively within their communities. Some participants reported they intentionally avoided high volume shopping hours and/or shopped in neighboring counties to avoid being recognized and/or minimize judgment from others.

### 4. Suggestions for Improving WIC Program and Its Services

Many suggestions were made by the participants on ways to improve their WIC experiences within three main topics: *improving the available food packages (*Theme 4A), suggestions for *enhanced nutrition education* services (Theme 4B), and suggestions for *expanded community outreach, knowledge, and awareness of the WIC program* (Theme 4C).

Participants reported receiving too much milk, yogurt, and/or cheese and cereal and thus frequently not fully utilizing these benefits (Theme 4A). General dissatisfaction with the juice benefit was apparent, with participants stating they would prefer to have more FV instead of juice. Greater flexibility in whole grains benefits was also desired (i.e. trade some cereal benefits for more bread or tortillas).

Enhancing and expanding nutrition education within the program also emerged as a suggestion for improving the WIC program and its services (Theme 4B). Participants expressed interest in post-partum weight loss education, reporting that they felt their needs were overlooked after their babies were born. Additional education on current food packages (i.e. existing flexibility of packages) and availability of various WIC-approved foods at different stores in each respective community was also suggested.

Lastly, *expanded community outreach, knowledge, and awareness* of the WIC program was an additional suggestion for improving the program (Theme 4C). General lack of awareness of WIC services was also reported. Many participants indicated they first heard about WIC by chance via word of mouth from someone who had personal experience using WIC, or at the local hospital after delivering a child. A few participants were aware of WIC services due to their college courses. Another major barrier to WIC program enrollment was confusion about eligibility criteria. Most participants did not know women might be eligible for WIC services starting during pregnancy, thus many of them did not enroll until after their first child was born, despite being aware of the WIC program. Confusion about income and/or other adjunctive eligibility criteria also emerged as major barriers to seeking enrollment (Table 2; see Additional Files). Participants felt strongly that additional WIC outreach efforts are needed to expand community awareness of the program.

## IMPLICATIONS

Even though WIC participation is associated with many improved health outcomes among women, infants, and children nationwide,^35^ little is known about maternal attitudes, barriers, and perceptions of the WIC experience of low-income families in rural areas of the U.S. Thus, the qualitative findings of the current study presented here help fill an important gap in the existing literature and provide a specific direction for practice and future research in this area.

Consistent with previous work,^20^,^23^,^36^ financial benefits were reported as a strong motivating factor for participating in the program, particularly for mothers receiving breastfeeding and formula benefit packages. Although long wait times and difficulty scheduling appointments have been cited as major barriers to program participation in other areas of the nation,^19^,^20^ participants in this study noted the efficiency of office visits and ease/flexibility of scheduling appointments as some of the most positive aspects of their WIC experience, stating they feel this is due to a more intimate, “small town” feel of the district. Interactions with program staff were reported to be mostly positive with participants citing staff generally had a caring and nurturing approach. However, a few negative aspects of the office experience were also identified. Most notably was the perception that staff can be “pushy” with providing nutrition and breastfeeding education. For example, participants perceived pressure about what, how, and when to feed certain foods to their children.

Some of the findings on barriers to WIC participation/benefit redemption are consistent with previous larger-scale investigations in various regions of the nation. Poor labeling of WIC-approved foods^19^,^20^,^23^,^37^ has been reported nationwide and was also extensively reported by participants in this study. In the current study, mothers reported frustration with a lack of variety and availability of WIC-approved items in their local stores, which was also cited by WIC participants in Mississippi.^20^ Rural grocery stores are generally smaller and thus offer less variety than stores found in urban communities.^38^ As such, it is not surprising the greatest lack of variety and availability of WIC-approved foods was cited by participants in the two most rural counties, which also have a limited number of grocery stores. This highlights the need for WIC agencies in small rural regions such as the Appalachian Region to establish and/or strengthen mutually beneficial partnerships with local WIC-vendors to incentivize stocking a greater variety of WIC-approved foods beyond the minimum inventory required by federal legislation. Recent efforts by the Healthy Retail Working Group, a joint collaboration of the Robert Wood Johnson Foundation’s Healthy Eating Research (HER) program and the Centers for Disease Control and Prevention’s Nutrition and Obesity Policy Research and Evaluation Network (NOPREN), represent a step in the right direction toward improving access to healthier foods and beverages for low-income, young children and their families.^39^ However, there is still great need for more targeted efforts among Appalachian families, particularly in Appalachian North Carolina, to address challenges and barriers accessing healthy foods.

The most pervasive barrier to participation reported in the current study was social stigma. In other larger studies, social stigma has been perceived as having a mild to moderate impact on participation,^19^,^20^,^23^,^37^,^40^ but was cited as a significant barrier to participation in this sample of WIC mothers in rural Appalachian North Carolina. Strikingly, social stigma was either explicitly mentioned or alluded to by all participants as a barrier to participation. While the “small town” feel of this district was cited as a positive aspect of the WIC experience, participates noted it also contributed to stronger perceptions of social stigma and embarrassment/shame. When asked to discuss their shopping experience and barriers related to participating in the WIC program, participants shared they have experienced both verbal and nonverbal signs of disapproval and judgment by other shoppers and cashiers while redeeming their WIC benefits in the local stores. Several participants shared personal stories of being identified as “one of them” by other shoppers or publicly criticized by store clerks for “wasting the government’s money.” Others expressed feelings of guilt and shame for accepting assistance when they also work a full-time job. As such, they suggested the feelings of embarrassment stemming from social stigma while shopping is likely a major deterrent to participation for some families in the region. These findings suggest mothers in this sample may perceive that the benefits of participation (i.e. food, breast pump) out-weigh the costs (i.e. social stigma, embarrassment/shame), confirming previous findings of such phenomenon.^20^,^23^

In this context, it is important to consider the influence of the unique culture of the Appalachian Region on the perceptions and experiences of social stigma. This region is largely conservative and known for valuing individualism and self-reliance as well as religious fundamentalism and fatalism.^41^,^42^ Widespread distrust of outsiders and government and general reluctance to change are additional core Appalachian values. Interestingly, these cultural norms were alluded to by women in this study as contributing factors to their perceptions of social stigma. It is apparent that Appalachian culture largely contributes to social stigma perceived by women who receive WIC benefits in this region, and personal values may be reflected in their perceptions of stigma and embarrassment/ shame. Future research should further examine psychosocial barriers to participation in this region and explore potential strategies to reduce community-level social stigma associated with participation in WIC and other federal assistance programs.

The confusion about eligibility criteria, a known barrier to WIC participation,^19^,^20^,^23^ was largely related to participants not knowing that pregnant women qualify for WIC benefits. The fact that no participants heard about WIC from a pediatrician’s or obstetrician’s office is concerning because healthcare providers should serve as a key source of information about the assistance programs in the community, especially in rural areas. A better understanding of what healthcare professionals in this region of Appalachia know about the WIC program and how they perceive WIC services is needed to identify more effective ways for pediatricians and obstetricians to make referrals of eligible families to the WIC program.

This study has several major strengths but also limitations that must be noted. A key strength is that to the authors’ knowledge, it is the first study of its kind to explore barriers and perceptions of the WIC experience from the perspective of WIC mothers and pregnant women in rural Appalachia. Further, recruitment of participants from a WIC agency that includes three bordering counties with stratified degrees of rurality allowed for the examination of a broader range of attitudes, barriers, and perceptions surrounding the WIC experience in rural Appalachia, as some experiences varied by county. The in-depth, qualitative nature of this study also allowed for mothers to share their own experiences in a focus group format, which allows for a deeper understanding of the WIC experience in this region. A few limitations should also be noted. First, because of resource limitations, non-English speakers were not included despite having some Hispanic families enrolled in WIC in the region. Secondly, mothers experiencing transportation barriers may not have signed up to participate. Third, the majority of participants in this study were non-Hispanic white and all participants had at least some college education, which may have influenced their decision to participate. Considering the majority of WIC participants nationwide have a high-school education,^43^ the perceptions and experiences of focus group participants in this study may not reflect the larger WIC population in this region.

The WIC program can be effective in reducing nutrition-related inequalities experienced by low-income families with young children in the Appalachian Region. However, our findings show that rural families have unique experiences when participating in WIC and are faced with specific barriers that need be addressed in order to increase WIC enrollment and reduce program attrition of families in this region. This study provides insight into the WIC experience in rural Appalachian North Carolina and lays the foundation for further investigation. More targeted efforts that take into consideration regionally inherent structural, cultural, and economic challenges are needed to fully maximize the broader societal benefits of WIC participation in the region. Specifically, WIC agencies should focus on enhancing and/or creating partnerships and collaborations with local pediatrician and obstetrician offices, religious organizations, food banks, and grocery stores to expand awareness and knowledge of WIC services while reducing social stigma among community members. Additional research is also warranted to fully understand perceptions and attitudes related to WIC’s mission among various community stakeholders that serve low-income families with young children in rural Appalachia.

**SUMMARY BOX**

**What is known about this topic?** Although participation in WIC has been associated with favorable nutrition-related health outcomes in low-income families, WIC-eligible families continue to face a number of barriers to participation.

**What is added by the report?** Lack of variety/availability of WIC-approved foods and social stigma were perceived as major barriers to participation and redeeming benefits among WIC participants in rural Appalachian North Carolina.

**What are the implications for future research?** Findings are valuable for informing WIC state-agencies and policymakers whose efforts focus on the identification and development of effective recruitment and retention strategies for WIC-eligible families in rural Appalachia. A better understanding of what healthcare professionals in this region of Appalachia know about the WIC program and how they perceive WIC services is needed to identify more effective ways to increase awareness and utilization of WIC services in this region.

## Figures and Tables

**Table 1:** Demographic Characteristics of Women Participating in WIC^a^ in Appalachian North Carolina

Participant & Household Characteristics	All Participants (n = 15)	County #1 (n = 6)	County #2 (n = 5)	County #3 (n = 4)

	**Mean (SD)**			

**Participant Age**	28.7 (7.9)	25.8 (5.3)	33.4 (11.8)	27.3 (2.5)

**Years Participating in WIC**	3.3 (3.1)	3.1 (3.6)	4.2 (3.5)	2.4 (2.4)

**No. Adults 18+ years**	**Count (%)**			

1 adult	2 (13)	1 (17)	1 (20)	0
2 or more adults	13 (87)	5 (83)	4 (80)	4 (100)

**No. Children 5 to 17 years**				
None	6 (40)	1 (17)	2 (40)	0
1 child	7 (47)	4 (66)	5 (40)	3 (75)
2 or more children	2 (13)	1 (17)	1 (20)	1 (25)

**No. Children < 5 years**				
None	2 (13)	2 (33)	0	0
1 child	9 (60)	1 (17)	4 (80)	4 (100)
2 or more children	4 (27)	3 (50)	1 (20)	0

**Participant BMI** b				

Overweight (25.0 – 29.9)	3 (25)	2 (50)	1 (20)	0
Obese (30.0 or greater)	9 (75)	2 (50)	4 (80)	3 (100)

**Participant Education**				
Some College	4 (27)	2 (33.3)	1 (20)	1 (25)
2-year College Degree	4 (27)	2 (33.3)	1 (20)	1 (25)
4-year College Degree	5 (33)	2 (33.3)	1 (20)	2 (50)
Greater than 4-year degree	2 (13)	0	2 (40)	0

**Participant Race/Ethnicity**				
Non-Hispanic White	14 (93)	6 (100)	5 (100)	3 (75)
Hispanic White	1 (7)	0	0	1 (25)

a WIC = Special Supplemental Nutrition Program for Women, Infants, and Children

b BMI data for 2 participants in County #1 and 1 participant in County #2 excluded because of pregnancy.

**Table 2:** Themes & Subthemes Identified Through Focus Groups About WIC^a^ Participants Experiences in Rural Appalachian North Carolina

Themes and Subthemes	Representative Quotes
**1. Most Valued Aspects of WIC Participation**	
Theme 1A. *Financial Benefits*	“I mean just probably $100 a month worth of savings if not more…then when you, if you supplement or whatever, the formula is also very helpful.”
	“They gave me the pump which was ecstatic because, those are expensive and I definitely couldn't afford one.”
Theme 1B. *Support/Resource Benefits*	“…So WIC was very instrumental in ….putting us in …connection with health related dietitians, and speech pathologist and, just other… people in our community that I had no idea where to turn to.”
	“[The nutritionist] has really helped me, with the breastfeeding, an' she's answered every question if she didn't know the answer to my question she would find out and she'll call me back.”
**2. Experiences During WIC Appointments**	
**Positive Aspects:** Theme 2A. *Efficiency of Clinic Visits*	“I do love how they get you in and get you out [so] quick.”
Theme 2B: *Caring and Nurturing Approach*	“We love to go to the WIC office…they spoil us, they take care of us, they're very kind… they're good with questions or concerns we have.”
**Negative Aspects** Theme 2C: *Discrepancy in Nutrition Recommendations from WIC staff and Pediatricians*	“[My baby] had a sore butt for like a week… And so the doctor was like, *‘Well try cutting out dairy.’* And so I did and her butt cleared up. I told [the nutritionist] that, and she was like, *‘Oh, the doctors always blame it on the dairy first and you shouldn't listen to what the doctor says.’”*
Theme 2D: *High-Pressure Approach*	“When you're parenting, you kinda take from like, a little bit from everybody and see what works with your child it seems like, right?.…But it just seems like the way that, that sometimes [the nutritionist] approaches [feeding recommendations], it's like, "No! This is like, this is just one way.”
Theme 2E: *High-Pressure Approach*	“With all three of my children, I have been made to publicly, breastfeed, in front of someone in the WIC office…I felt like I had to prove that I was breastfeeding.”
**3. Barriers Related to Redeeming Benefits**	
Theme 3A. *Poor Labeling in Stores*	“[The grocery store] does not label things very well at all. Apparently, I just found out this like last week, they have, sometimes they don't have the big blue label that says WIC, but you can look somewhere in the corner, there is one little W which means it is WIC approved.”
Theme 3B. *Problems Redeeming WIC Approved Items*	“Like, like with brands and stuff like that, like [Store 1] will say one thing, [Store 2] will say another. Um, and I don't know if you can get Reese’s peanut butter at [Store 2], but you can at [Store 1].”
Theme 3C. *Lack of Variety of WIC Approved Foods Locally Available*	“…locally the store only stocks one of these five or six breads, so I don't even bother looking at the others.”
Theme 3D. *Limited Number of Grocery Stores*	“You have 2 options in this entire town to shop for WIC at [Store 1] or at [Store 2] an' at [Store 2] at only one register.”
	“I recently went to [a grocery store in a larger county], and I was just amazed at how large their produce and vegetable section was, ours is like an eighth of the size. Like it was just amazing, to walk through. I think that's a big disadvantage just overall is we don't have as big a selection.”
Theme 3E. *Delays at Checkout*	“Even goin' to the checkout, like [Speaker 302] said, it's just like, you almost dread it because it was like you do take a long time.”
Theme 3F. *Social Stigma*	“Just their body language, lets you know that they're communicating their judgment, towards you. And they don't even have to say a word.”
	“Even goin' to the checkout, … it's just like, you almost DREAD it because it was like you do take a long time and people get behind you, you're like *"Oh man!"* And then like, even the cashiers, like, you know, they just kind of like judge you … sometimes it was just embarrassing, and like I even had like some cashiers like visually say like, ‘*Oh my God!’*"
**4. Suggestions for Improving WIC Program and Services**	
Theme 4A. *Available Food Packages*	“We get allotted too much [juice], um, you know, we're encouraged not to give our children, those sort of products, but, at the same time, that's what we're approved for.”
	“If they could add maybe some of the store brands of the whole wheat pasta. That would be wonderful because then I could, could use that.”
Theme 4B. *Enhanced Nutrition Education Services*	“I would like to have more information on postpartum weight loss.”
	“Maybe um, explaining what your options are. So I've never been, no one ever explained to me, from, the office, that I could switch yogurt for milk or cheese for milk or, stuff like that, or, what you were getting.”
	“You offer [a] nutrition program and you're not educated really on the best way to use it.”
Theme 4C. *Expanded Community Outreach, Knowledge, and Awareness of WIC Program*	“My sister lives… [in] a bigger place, an' she told me about [WIC]. I felt like I'm, like I haven't heard about [WIC] around here like nobody really talks about it around here.”
	“I wasn't even aware of [WIC] as a, um, member of the community an' even as a foster parent until my children came to me as foster children and immediately DSS made me aware of it.”
	“I started [using WIC] toward the end of my first pregnancy because I didn't know that you could be pregnant and get WIC.”

a WIC = Supplemental Nutrition Program for Women, Infants, and Children’s Program

WIC Program Experiences and Barriers in Rural Appalachian North Carolina
